# Can evolutionary therapy be applied in non-small cell lung cancer?

**DOI:** 10.1038/s41598-026-36712-x

**Published:** 2026-02-05

**Authors:** Laura R. Jansén-Storbacka, Kailas S. Honasoge, Eva Molnárová, Arina Soboleva, Bram C. Agema, Marthe S. Paats, Dirk Jan A. R. Moes, G. D. Marijn Veerman, Alethea B. T. Barbaro, Roel Dobbe, Irene Grossmann, Sepinoud Azimi, Ron H. J. Mathijssen, Anne-Marie C. Dingemans, Kateřina Staňková

**Affiliations:** 1 Institute for Health Systems Science, Delft University of Technology, Faculty of Technology, Policy and Management, Delft, The Netherlands; 2 Department of Respiratory Medicine, Erasmus Medical Center Cancer Institute, Rotterdam, The Netherlands; 3 Department of Medical Oncology, Erasmus Medical Center Cancer Institute, Rotterdam, The Netherlands; 4 Department of Clinical Pharmacy and Toxicology, Leiden University Medical Center, Leiden, The Netherlands; 5 Delft Institute of Applied Mathematics, Delft University of Technology, Delft, The Netherlands

**Keywords:** Mathematical oncology, metastatic cancer, PK/PD, evolutionary therapy, Zhang et al.’s protocol, non-small cell lung cancer, treatment-induced resistance, Cancer, Computational biology and bioinformatics

## Abstract

Evolutionary therapy (ET) applies principles of evolutionary biology to steer tumour dynamics and forestall or delay treatment resistance, typically guided by data-driven mathematical models. Our aim is to assess whether ET protocols, and specifically Zhang et al.’s protocol proposed for metastatic castrate-resistant prostate cancer, can be theoretically effective for fast-growing metastatic cancers such as stage IV non-small-cell lung cancer (NSCLC). Using longitudinal tumour-burden data from NSCLC patients treated with erlotinib, we systematically evaluate 26 two-population differential-equation models based on classical tumour-growth dynamics, with varying assumptions about density- and frequency-dependent interactions, pharmacokinetics, and treatment-induced death. Previous work by Yin et al. on the same dataset employed an exponential model that omitted density- and frequency-dependent interactions; although it provided a good fit to tumour-burden data, its structure would theoretically lead to poorer outcomes under ET protocols. In contrast, our analysis identifies the minimal model structure required to reproduce the resistance-driven regrowth observed in NSCLC, with the Gompertzian model featuring log-kill dynamics and both density- and frequency-dependent interactions providing the best fit. In this model, Zhang et al.’s protocol prolonged median time-to-progression to 42.3 months compared with 24.8 months under maximum tolerated dose. These results indicate that ET is theoretically a viable treatment strategy for NSCLC. This study offers a practical framework for assessing ET feasibility using clinical data and supports future clinical translation of ET in NSCLC.

## Introduction

Resistance to therapy remains one of the most formidable challenges in the treatment of metastatic cancer. While many tumours initially respond to therapy, sustained responses are rare due to the evolution of drug-resistant cell populations. This is particularly problematic in metastatic disease, where tumour heterogeneity and evolutionary pressures are prevalent^1^. Traditional treatment protocols that rely on maximum tolerated dose (MTD) often accelerate resistance and compromising long-term outcomes^2–9^. Moreover, MTD-based regimens frequently reduce patient quality of life due to high drug toxicity.

In metastatic non-small cell lung cancer (NSCLC), the leading cause of cancer-related death worldwide^2^, the introduction of tyrosine kinase inhibitors (TKIs) has markedly improved survival in patients with actionable driver mutations^10^. Erlotinib, a first-generation EGFR-TKI, is commonly used in this context, but resistance to therapy eventually develops in nearly all patients^11,12^. This challenge has motivated the exploration of alternative dosing strategies that can delay resistance evolution while reducing toxicity. Intermittent and metronomic regimens have shown promise in some cancers, such as prostate and melanoma^13–15^, and improved quality of life in elderly NSCLC patients treated with chemotherapy^16^. Preclinical studies in NSCLC have also shown that resistance to erlotinib may decline in drug-free conditions^17^, and pulsed high-dose regimens do not increase toxicity in phase I trials^18^.

One promising alternative to standard of care is *evolutionary therapy*, which applies principles from evolutionary biology to adjust treatment in response to tumour behaviour^19–25^. Rather than aiming for maximal cell-kill, evolutionary therapy typically seeks to exploit ecological interactions among cancer cell populations, such as competition between drug-sensitive and resistant cells, to delay resistance and prolong tumour control^25,26^. A pioneering example of this approach is the trial by Zhang et al. in metastatic castrate-resistant prostate cancer, where adaptive on–off abiraterone dosing significantly prolonged time to progression^27–30^. This success has sparked interest in extending evolutionary protocols to other cancer types^28,31,32^. Its applicability to metastatic NSCLC remains unexplored, in part due to challenges in biomarker availability^26,33^, and the aggressive nature of NSCLC. In this context, mathematical models provide a valuable tool to explore and optimise evolutionary therapies in silico before clinical testing^3,20,24,34–36^. They enable comparison of different dosing strategies and how they affect clinical outcomes.

A recent application of mathematical modelling to NSCLC was in a study by Yin et al., where a variant of the polymorphic exponential model was fitted to longitudinal tumour burden data from NSCLC patients treated with erlotinib^37^. Their model includes drug-sensitive and drug-resistant cancer cell populations and assumes a constant, treatment-independent mutation rate from sensitive to resistant cells. It does not, however, incorporate density-dependent or frequency-dependent interactions, which have been shown to be key factors in cancer growth and evolution^38–41^.

In this study, we investigate whether a specific ET protocol could theoretically work in patients with metastatic NSCLC. We choose the protocol of Zhang et al. as it is the best known ET protocol and has already been successfully trialled^28^. Unlike Yin et al.’s exponential model, which assumes no ecological interactions, we investigate a broader class of two-population models that explicitly include density- and frequency-dependent competition. To do this, we systematically validate 24 alternative two-population models based on classical cancer dynamics^42,43^, using the same dataset analysed by Yin et al., and identify which models best capture the dynamics of metastatic NSCLC under erlotinib.

We then use the best-fitting models to simulate the evolutionary therapy protocol of Zhang et al.^27^, which uses on–off dosing to maintain competition between the two cell types, and we evaluate its predicted impact on time to progression (TTP), defined as the time until tumour burden exceeds 1.1$\times$ its initial value-a common proxy in ET studies.

## Results

### Mathematical models

#### Population growth models

We consider three base models for the population growth of sensitive cells (with population $x_S(t)$ at time *t*) and resistant cells (with population size $x_R(t)$ at time *t*): the Logistic model, the Gompertzian model, and the von Bertalanffy model^42,44^. In all of these models, $r_S$ and $r_R$ refer to the growth rate of the sensitive and resistant cancer populations, respectively, $\alpha _{RS}$ refers to the competition effect of sensitive cells on the resistant population, $\alpha _{SR}$ refers to the competition effect of the resistant cells on the sensitive population, and *K* serves as a carrying capacity. These parameters are fitted to longitudinal volumetric measurement data as described in the section titled ‘Data Sources and Processing’ in the Methods. For all models, the sensitive population $x_S$ grows as long as $x_S + \alpha _{SR}\, x_{R} < K$, and the resistant population grows as long as $\alpha _{RS}\, x_S + x_{R} < K$. If $\alpha _{RS}=\alpha _{SR} = 1$, then growth depends only on the total cancer population. However, when these parameters are not fixed to 1,  frequency dependence arises, which means that the growth of each cell type depends on the relative frequencies (or ratios) of the cell types within the population. The Logistic two-population model is given by

1 $$\begin{aligned} {\left\{ \begin{array}{ll} \dot{x}_S(t) = r_{S}\, x_{S}(t) \left( 1-\frac{x_S(t) + \alpha _{SR}\, x_{R}(t)}{K}\right) ,\\ \dot{x}_R(t) = r_{R}\, x_{R}(t) \left( 1-\frac{\alpha _{RS}\, x_S(t) + x_{R}(t)}{K}\right) . \end{array}\right. } \end{aligned}$$

The Gompertzian two-population model takes the form

2 $$\begin{aligned} {\left\{ \begin{array}{ll} \dot{x}_S(t) = r_{S}\, x_{S}(t)\, \ln \left( \frac{K}{x_S(t) + \alpha _{SR}\, x_{R}(t)}\right) ,\\ \dot{x}_R(t) = r_{R}\, x_{R}(t) \, \ln \left( \frac{K}{\alpha _{RS}\, x_S(t) + x_{R}(t)}\right) . \end{array}\right. } \end{aligned}$$

The third model that we consider, the two-population von Bertalanffy model^45,46^, is given by

3 $$\begin{aligned} {\left\{ \begin{array}{ll} \dot{x}_S (t) = r_{S} \, \left( x_{S}(t)\right) ^{{2}/{3}} \, \left( 1- \frac{\left( x_S (t) + \alpha _{{SR}}\, x_{R}(t)\right) ^{{1}/{3}}}{K}\right) ,\\ \dot{x}_R (t) = r_{R} \, \left( x_{R}(t)\right) ^{{2}/{3}} \, \left( 1- \frac{\left( \alpha _{RS}\, x_S(t) + x_{R}(t)\right) ^{{1}/{3}}}{K}\right) . \end{array}\right. } \end{aligned}$$

#### Density and frequency dependence

We include two types of competition in our models: density-dependent and frequency-dependent competition. Density-dependent competition arises when cells compete for space and/or limited resources. This is modelled using the carrying capacity *K*. All our models include density-dependent competition. Frequency-dependent competition, on the other hand, depends on the relative abundance of different populations. This means the impact of one population on the other’s growth depends on their relative proportions. Frequency-dependent competition is included in our models by using the competition coefficients $\alpha _{SR}$ and $\alpha _{RS}$ to quantify how strongly the populations influence each other’s growth. For each growth model, we fit two different versions: a purely density-dependent model, where we set $\alpha _{RS} = \alpha _{SR} = 1$, and a frequency-dependent model, where in addition to the density dependence, we include frequency dependence by modelling $\alpha _{RS}$ and $\alpha _{SR}$ as free parameters which are estimated from the data.

#### Pharmacokinetics (PK) and pharmacodynamics (PD)

Following Yin et al.^37^, we choose a linear PD model, i.e., the effect of the medication increases linearly with the treatment dose^47^. Treatment-induced cell death depends on how much treatment reaches cancer cells. Plasma drug concentration varies between treatment doses. Generally, in PK modelling, an exposure metric, $E_{\text {metric}}$, such as area under the concentration-time curve (AUC), maximum or minimum observed concentration ($C_{\text {max}}$ or $C_{\text {min}}$), lowest concentration observed before the next dose ($C_{\text {trough}}$), or the dose (*m*(*t*)) is used^48,49^. These are examples of time-aggregated metrics where the dynamics of the drug concentration are ignored and information is condensed into a single number. We refer to such models as aggregated-PK (agPK) models.

Alternatively, a time-varying metric may be used, such as the concentration predicted by a PK model fitted to PK data^48,49^. In this case, the concentration dynamics are accounted for and affect the tumour growth. We term such models dynamic-PK (dyPK) models. Here, we used a two-compartment model with first-order absorption and linear elimination similar to Yin et al. ^37^ (see Appendix 2 for detailed explanation).

In our models, we define a medication function *c*(*t*) that captures both the above-mentioned cases:

4 $$\begin{aligned} c(t) {\mathop {=}\limits ^\textrm{def}} {\left\{ \begin{array}{ll} m(t) \, D_{MTD} \, {s_{NP}},\quad \quad \quad \quad \text{ for } \text{ agPK } \text{ models }, \\ m(t)\, D_{MTD} \, C(t) \, {s_{P}},\,\quad \,\,\quad \,\text{ for } \text{ dyPK } \text{ models }, \end{array}\right. } \end{aligned}$$

with the maximum tolerable dose $D_{MTD}$ defined in milligrams (*mg*). The physician’s decision is captured by the medication ratio *m*(*t*) at time *t* and lies in the interval [0, 1],  with $m(t)=0$ and $m(t)=1$ corresponding to no treatment and maximum tolerable dose, respectively, and *C*(*t*) gives the plasma concentration normalised to the treatment dose $m(t) \cdot D_{MTD}$ at time *t* (see Appendix 2 for its definition). The parameters $s_{NP}=1$ and $s_{P}=1000$ are scaling factors with units $mg^{-1}$ and $l\cdot \mu g^{-1},$ respectively. In the rest of this paper, comparisons of the agPK and dyPK models are made. It is important to note that the results for the agPK models are independent of the choice of exposure metric $E_{\text {metric}}$. In other words, if we replace the treatment dose ($m(t) \cdot D_{MTD}$) at time *t* by, for instance, AUC or $C_{\text {min}}$, the results will not change.

#### Treatment-induced death rate

We consider two standard assumptions regarding treatment-induced death of treatment-sensitive cancer cells: the log-kill (LK) model and the Norton-Simon (NS) model^50–53^. With the log-kill treatment-induced death rate, the population of sensitive cancer cells killed at time *t* is $\lambda \cdot c(t),$ with $\lambda >0$ denoting the treatment effect, a parameter to be fitted. Hence, for example, the Logistic model with the LK treatment-induced death rate becomes

5 $$\begin{aligned} {\left\{ \begin{array}{ll} \dot{x}_S(t) = r_{S}\, x_{S}(t) \left( 1-\frac{x_S(t) + \alpha _{SR}\, x_{R}(t)}{K}\right) - \lambda \, x_{S}(t)\, c(t),\\ \dot{x}_R(t) = r_{R}\, x_{R}(t) \left( 1-\frac{\alpha _{RS}\, x_S (t) + x_{R}(t)}{K}\right) . \end{array}\right. } \end{aligned}$$

A key principle of the NS hypothesis is that the treatment-induced death rate is proportional to the sensitive cells’ growth rate. The effect of treatment is thus incorporated by multiplying the right-hand side of the evolution equation for $x_S$ by $(1 - \lambda c(t)),$ where $\lambda$ represents the treatment effect and the medication function $c(t)$ defined in (4).

As an example, applying this modification to the Logistic growth model yields the following system:

6 $$\begin{aligned} {\left\{ \begin{array}{ll} \dot{x}_S(t) = r_{S}\, x_{S}(t)\, \left( 1-\frac{x_S(t) + \alpha _{SR}\, x_{R}(t)}{K}\right) \left( 1-\lambda c(t)\right) ,\\ \dot{x}_R(t) = r_{R} x_{R}(t) \,\left( 1-\frac{\alpha _{RS}\, x_S (t) + x_{R}(t)}{K}\right) . \end{array}\right. } \end{aligned}$$

We consider three growth models, two alternative assumptions regarding frequency dependence, two alternative assumptions regarding the pharmacokinetics, and two different treatment-induced death rate functions, giving 24 two-population models. We compare the ability of these models to capture NSCLC dynamics under erlotinib treatment to that of the polymorphic exponential model^37^ given by

7 $$\begin{aligned} {\left\{ \begin{array}{ll} \dot{x}_{S}(t) = \left( r -\mu \right) \, x_{S}(t) - {\lambda }\,x_S(t) \, c(t),\\ \dot{x}_{R}(t) = r\, x_{R}(t) + \mu \cdot x_{S}(t). \end{array}\right. } \end{aligned}$$

The polymorphic exponential model assumes a single growth rate *r*, i.e., $r_S=r_R=r$. The expression $\lambda \cdot c(t)$ denotes treatment-induced death rate of sensitive cancer cells, with parameter $\lambda$ and medication function *c*(*t*),  defined by (4). It also assumes a mutation rate $\mu$ of sensitive cells into resistant cells, independent of medication. For this model we also consider two alternative assumptions for pharmacokinetics. This model has been previously fitted^37^ to the same dataset that we use for our simulations.

Table 1 summarises the key variables and parameters of the 24 models we propose, as well as those of the polymorphic exponential model.

**Table 1:** Descriptions of key parameters and variables used in our models together with their units. Not all parameters are included in every model.

Parameter	Description	Units
$r_{S}$	The intrinsic growth rate of sensitive cells	day$^{-1}$
$r_{R}$	The intrinsic growth rate of resistant cells	day$^{-1}$
*r*	Shared growth rate of sensitive and resistant cells (polymorphic exponential model)	day$^{-1}$
$\lambda$	Effect of medication of dose *m*(*t*) on sensitive cell growth	mg$^{-1}$day$^{-1}$
*K*	Carrying capacity of the total cancer cell population	mm$^3$
$\alpha _{SR}$	Competitive effect of resistant cells on sensitive cells	dimensionless
$\alpha _{RS}$	Competitive effect of sensitive cells on resistant cells	dimensionless
$\mu$	Mutation rate of sensitive cells into resistant cells (polymorphic exponential model)	day$^{-1}$

Variable	Description	Units
$x_{S}(t)$	Size of the sensitive cell population at time *t*	mm$^3$
$x_{R}(t)$	Size of the resistant cell population at time *t*	mm$^3$
$D_{MTD}$	Maximum tolerable dose for a given drug	mg
*D*(*t*)	Dose administered at time *t*	mg
*m*(*t*)	Treatment dose ratio: $\frac{D(t)}{D_{MTD}}$	dimensionless

### Models using the log-kill death rate fit better than those using the Norton-Simon death rate

To fit model parameters to individual patient data, we used differential evolution, a population-based heuristic optimisation algorithm for non-linear problems. The method iteratively improves candidate solutions through mutation, crossover, and selection, and is often considered less sensitive to premature convergence to local minima than gradient-based approaches^54,55^.

We fit the model parameters both individually, where each patient has unique fitted parameters, and population-wide, where the same parameter values are fitted for all patients. For individual fits, we minimised the relative percentage error, whereas for population-wide fits, we minimised the average relative percentage error across all patients.

There is a considerable difference in the goodness of fit of models with different treatment-induced death rates. Figure 11 in Appendix 4 shows that the LK death rate decreases the relative percentage error of individual fits for all models compared to the NS death rate. A similar effect is observed for population-wide fits, where acceptable goodness of fit is achieved only by the Gompertzian and Logistic models with a log-kill treatment-induced death rate (Table 2). Given these results, we continue analysing only models with the log-kill death rate in the rest of the study.

### Pharmacokinetic type does not impact model goodness of fit

**Fig. 1:**
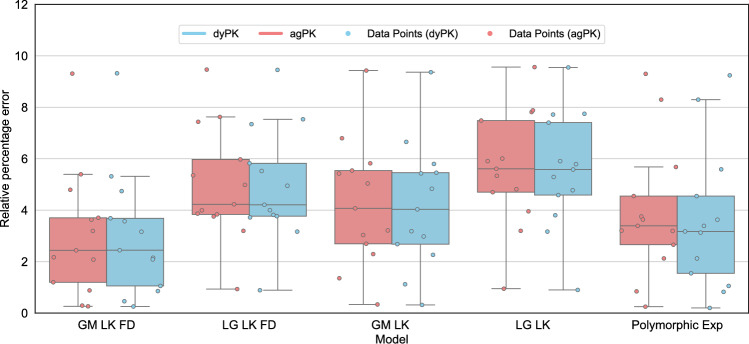
Relative percentage error of individually-fitted models for the best performing models. The Gompertzian model with frequency dependence demonstrates the best fit. Abbreviations: GM - Gompertzian, LG - Logistic, Polymorphic Exp - Polymorphic Exponential Model, LK - log kill, FD - Frequency dependence. Two types of pharmacokinetics, dynamic pharmacokinetics (dyPK) and aggregated pharmacokinetics (agPK) are shown for each model.

We observed that the choice of pharmacokinetic modelling type, aggregated pharmacokinetics (agPK) or dynamic pharmacokinetics (dyPK), did not significantly affect the goodness of fit. Among the five best-fitting models, only the poorest-fitting one (the Logistic model with log-kill dynamics and without frequency dependence, LG_LK) showed a statistically significant difference in paired t-tests (see Table 7 and Fig. 1). The measured effect was less than 0.1%, suggesting that the difference in goodness of fit is negligible in practical terms. This indicates that, from the perspective of goodness of fit, both pharmacokinetic types are equally suitable. Likewise, on a population-wide level, the best-fitting models show little to no difference in relative percentage error between the two pharmacokinetic types (Tables 2 and 7). Boxplots illustrating the distribution of relative percentage errors for the five models with the best individual-level fits are shown in Fig. 1, while boxplots for all models are provided in Appendix 4. The polymorphic exponential model no longer provides an adequate fit, whereas the other four top-performing models maintain good and consistent fits (Table 2).

**Table 2:** Population-wide fits for all models with treatment-induced death rate. Log-kill treatment-induced death rate improves fit for all models. For each model we compared dynamic pharmacokinetics (dyPK) with aggregated pharmacokinetics (agPK). The type of pharmacokinetics has little effect on the average relative percentage error (ARPE). Parameter boundaries are provided in Table 3.

Model type	Frequency dependence	Pharmaco-kinetics	ARPE with Log-kill	ARPE with Norton-Simon
Gompertzian	$\checkmark$	agPK	5.57	19.45
	$\checkmark$	dyPK	5.66	19.45
	-	agPK	6.67	20.71
	-	dyPK	6.67	20.71
Logistic	$\checkmark$	agPK	7.17	13.15
	$\checkmark$	dyPK	7.17	13.16
	-	agPK	7.18	18.56
	-	dyPK	7.18	18.56
von Bertalanffy	$\checkmark$	agPK	14.59	14.41
	$\checkmark$	dyPK	14.61	14.41
	-	agPK	14.61	14.41
	-	dyPK	14.61	14.41
Exponential	-	agPK	14.38	n.a.
	-	dyPK	14.38	n.a.

### The polymorphic exponential model is not suitable for predicting disease progression or guiding evolutionary therapy

The populations of sensitive and resistant cancer cells in the polymorphic exponential model at any time *t* can be calculated analytically (see Appendix 3):

$$\begin{aligned} x_S(t)&=x_S(0) e^{(r-\lambda -\mu )t},\\ x_R(t)&= x_S(0)\left( \frac{-\mu }{\lambda +\mu }\right) e^{(r-\lambda -\mu )t}+ x_S(0)\left( k+\frac{\mu }{\lambda +\mu }\right) e^{rt}. \end{aligned}$$

Here, *r* is the growth rate for the sensitive and resistant populations (denoted $x_S$ and $x_R$, respectively), $\lambda$ is the effect of medication on the growth of the sensitive cell population, $\mu$ is the mutation rate, and *k* is the ratio between the initial resistant and sensitive populations; refer to Table 1 for parameter definitions. It can clearly be seen from the equations in Appendix 3 that the size of the resistant population is entirely independent of whether any treatment has been given. Referring back to the model given by (7), we see that the mutation rate is simply directly proportional to the sensitive population.

Moreover, in the same appendix we show that the time *t* at which the tumour burden reaches any proportion *p* of the initial tumour burden satisfies

$$\begin{aligned} p&= \frac{1}{1+k}\left( e^{(r-\lambda -\mu )t} - \frac{\mu }{\lambda +\mu }e^{(r-\lambda -\mu )t}+\left( k+\frac{\mu }{\lambda +\mu }\right) e^{rt}\right) , \end{aligned}$$

a quantity independent of the initial tumour burden. This implies that the time before exceeding any given progression threshold of the polymorphic exponential model is a function of the parameters and independent of the initial tumour burden. For population-wide fits, the parameters are identical for all patients, meaning all patients progress at the same time. Since the mutation rate is proportional to the sensitive population and independent of the medication, when medication is paused and the sensitive population increases, this in turn causes the growth rate of the resistant population to increase. The absence of a competitive effect and the medication-independent mutation rate mean that the model may predict worse results under evolutionary therapy compared to MTD. Although the polymorphic exponential model can capture tumour burden data relatively well for many patients (Appendix 4), the mathematical properties discussed above disqualify its further usage for treatment optimisation.

**Fig. 2:**
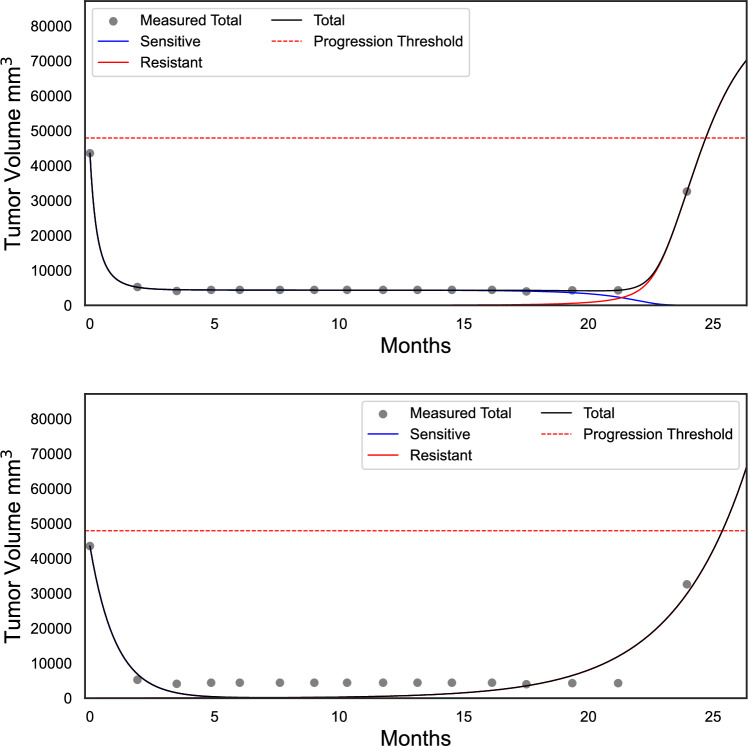
Fitting the tumour-burden data of patient 1002 with the Gompertzian model (above), which includes a log-kill treatment-induced death rate and frequency-dependent competition, and with the polymorphic exponential model (below). The progression threshold was set at 110% of the initial tumour burden. The Gompertzian model provides a better fit for this U-shaped trajectory.

### The Gompertzian model with frequency dependence most accurately describes NSCLC treatment dynamics

For both individual and population-wide fits, the Gompertzian model with frequency dependence has the best relative percentage error (Fig. 1 and Table 2). Moreover, of all the models tested, this model is best able to capture the U-shaped trajectory typical for treatment-induced resistance (Fig. 2).

We, therefore, proceed with the Gompertzian model with frequency dependence for simulations of evolutionary therapy. We select Zhang et. al’s therapeutic protocol, which aims to keep the tumour burden below the progression threshold by strategically switching the treatment on and off, allowing the sensitive cell population to regrow in order to keep the resistant population in check^27^. For comparison, we also simulate evolutionary therapy on the other best-fitting models and show this alongside the polymorphic exponential model (Fig. 1).

### The best-fitting models predict increased time to progression with Zhang et al’s protocol

**Fig. 3:**
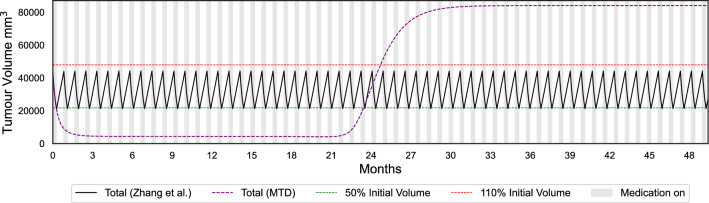
Comparison of predicted tumour growth for patient 1002 under evolutionary therapy (implemented using the Zhang et al.’s protocol) and MTD dosing regimens, using the Gompertzian model with frequency dependence.

Our simulations using the fitted parameters show that Zhang et al.’s protocol increases time to progression (defined as 1.1 times the initial tumour burden) when compared to the standard of care. We employ simulations to demonstrate the size of the potential benefit of Zhang et al.’s protocol for patients treated with erlotinib. As an illustrative example, we show the Gompertzian model with frequency dependence fitted to one patient in Fig. 3.

**Fig. 4:**
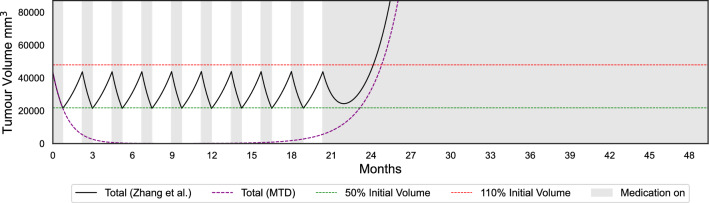
Predicted tumour growth for patient 1002 under evolutionary therapy (Zhang et al.’s protocol) and MTD dosing regimens using the polymorphic exponential model.

**Fig. 5:**
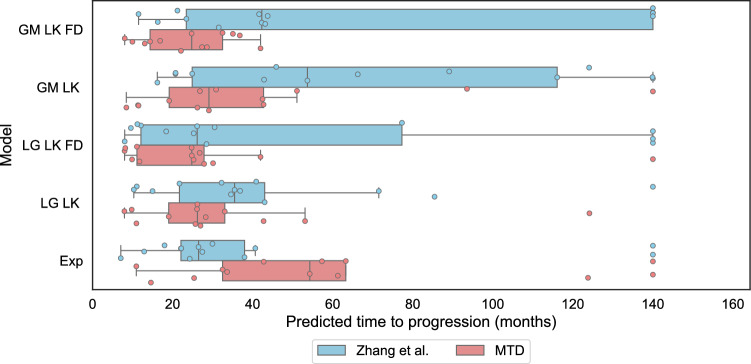
Predicted times before the tumour burden exceeds 1.1 times its initial size for the best individual fitting models under the Zhang et al.’s protocol and under the standard of care (MTD). The model names key is GM: Gompertzian, LG: Logistic, LK: log-kill, FD: with frequency dependence, Polymorphic Exp: polymorphic exponential model. Simulations were right-censored at 140 months, regardless of whether progression had occurred.

Figures 3 and 4 demonstrate expected time to progression (defined as 1.1 times the initial tumour volume) with MTD and Zhang et al.’s evolutionary therapy with the Gompertzian and Yin et al.’s models, respectively, for one of the erlotinib patients. While both models predict similar growth dynamics under MTD, they predict very different outcomes under an evolutionary therapy protocol. Similarly to the Gompertzian model, all best-fitting models presented in Fig. 1 predict a qualitatively similar benefit of Zhang et al.’s evolutionary therapy when compared to standard of care, as shown in Fig. 5. The simulations in Fig. 5 were continued beyond the initial span of the data. If the simulated trajectories had not reached the progression threshold during the simulated time, the data were right censored. In the Gompertzian and Logistic models, censoring occurred only during the simulations of evolutionary therapy as the dynamics for some patients allowed multiple repeated cycles. In contrast, when simulating with the polymorphic exponential model, censoring was also required for predictions under MTD, as the model predicted very slow long-term growth.

The median predicted TTPs for both MTD and Zhang et al.’s evolutionary therapy in models with frequency dependence (FD) are lower than those in models without FD (Fig. 5). Models with FD also exhibit lower RMSE values and fit the data more accurately (Fig. 1). This is expected since these models include two additional parameters compared to models without FD. For all patients, the optimised parameters in both the Gompertzian and logistic models with FD consistently satisfied $\alpha _{SR} \ne \alpha _{RS}$, indicating asymmetric competitive effects. In both models, the change in predicted TTP between models with and without FD depends on the ratio of the competition coefficients $\frac{\alpha _{SR}}{\alpha _{RS}}$. For instance, in the Gompertzian models GM_LK_FD (with FD) and GM_LK (without FD), all patients with $\frac{\alpha _{SR}}{\alpha _{RS}} < 1$ exhibited higher predicted TTP under the Zhang et al.’s protocol in GM_LK_FD than in GM_LK. For patients with $\frac{\alpha _{SR}}{\alpha _{RS}} > 1$, the opposite was observed. The median TTP decreases in both Gompertzian and logistic models when FD is included because the majority of patients in our dataset (approximately 77% for Gompertzian and 69% for logistic) had $\frac{\alpha _{SR}}{\alpha _{RS}} > 1$.

Additionally, the TTP gains achieved with Zhang et al.’s evolutionary therapy compared to MTD were greater for Gompertzian models than for logistic models (Fig. 5). This is consistent with previous theoretical studies showing that the reduction in growth rate for a marginal increase in total tumour burden is greater in the Gompertzian than in the logistic model of tumour growth^34,56^. Consequently, competitive effects are stronger in Gompertzian models, resulting in larger TTP gains than in logistic models.

**Fig. 6:**
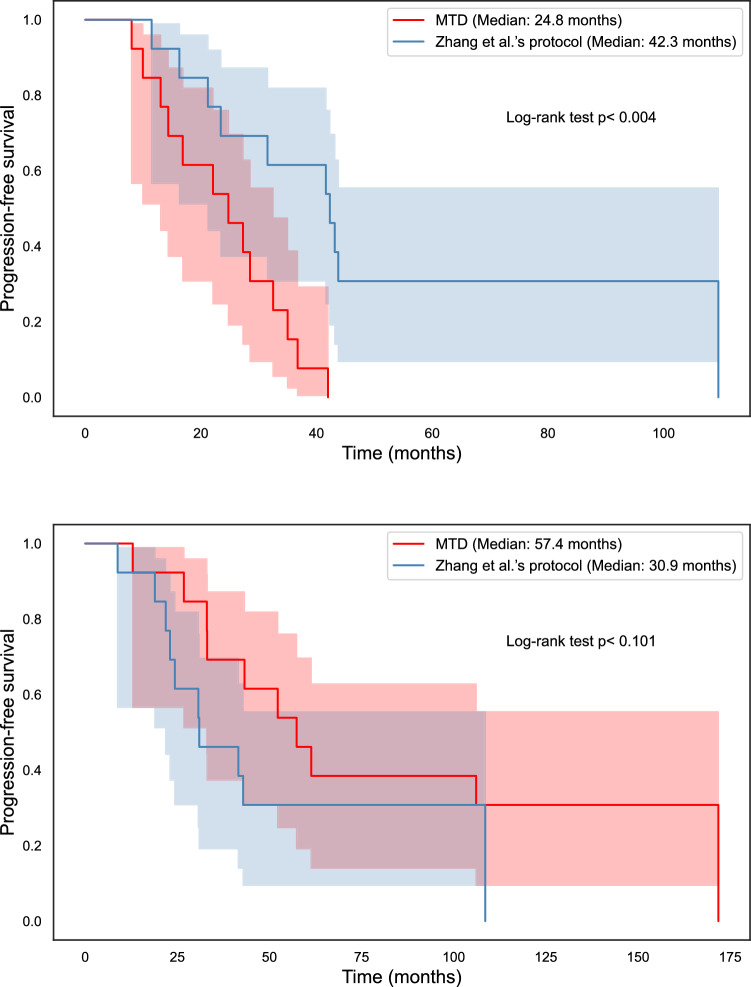
Kaplan-Meier plots of simulated progression-free survival over time for both MTD and Zhang et al.’s evolutionary protocol, for individually fitted Gompertzian model with frequency dependence, log-kill rate and agPK (above) and the polymorphic exponential model (below). The Gompertzian model and all other best-fitting models from Fig. 1 show improved time to progression (exceeding 1.1 times the original tumour burden) under Zhang et al.’s protocol, while the polymorphic exponential model predicts the opposite.

**Fig. 11:**
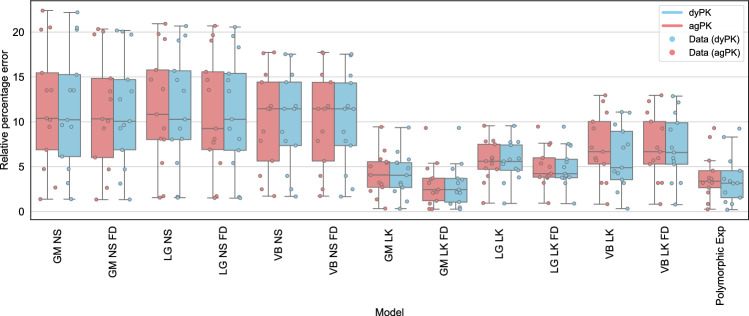
Box plots showing relative percentage error at the individual level for all tested models. The model key is GM: Gompertzian, LG: Logistic, VB: von Bertalanffy, Exp: polymorphic exponential, LK: log kill, NS: Norton-Simon kill, FD: with fitted competition coefficients.

Figure 6 presents Kaplan–Meier plots of the expected time to progression under Zhang et al.’s evolutionary therapy and MTD for all patients, for the Gompertzian and polymorphic exponential models, respectively. The Gompertzian model with frequency-dependent competition predicts a substantial benefit of evolutionary therapy, with the median time to progression increasing from 24.8 months under MTD to 42.3 months, an improvement of 17.5 months. All well-fitting models that include competition show a similar benefit. Kaplan–Meier plots for the other well-fitting models, and are provided in Appendix 7. In contrast, the polymorphic exponential model, which lacks both density- and frequency-dependent competition, predicts the opposite trend. It also suggests that some patients do not progress at all under MTD during the simulated time frame, which occurs when a high fitted mutation rate is offset by a very low growth rate of resistant cells. Figure 4 alsor illustrates that the absence of competition in the polymorphic exponential model leads to worse outcomes under Zhang et al.'s evolutionary therapy, reinforcing its unsuitability for ET protocol design (see Appendix 3 for analytical justification).

## Discussion

This study evaluated the potential of evolutionary therapy for non-small cell lung cancer (NSCLC) using mathematical models fitted to tumour burden data from patients treated with erlotinib. We first evaluated the goodness of fit of multiple two-population models, at both the individual and population levels, to identify those that best described the observed tumour dynamics. The models that provided the best fits were then used to simulate Zhang et al.’s evolutionary therapy protocol^27^. Time to progression, defined as the time when tumour burden exceeds 110% of its initial value, was compared between this protocol and standard-of-care treatment.

Our analysis showed that although several models can achieve a satisfactory goodness of fit to patient data, their ability to describe tumour dynamics and resistance evolution varies substantially. Among the fitted models, the two-population Gompertzian model with frequency dependence provided the best overall goodness of fit, capturing tumour-burden trajectories in metastatic NSCLC patients treated with erlotinib more accurately than alternative models. This emphasises the importance of including parameters that represent density-dependent interactions as well as frequency-dependent interactions between cancer cell types, when developing models to optimise systemic therapies. This is consistent with previous in-vitro studies^38,39^ demonstrating frequency-dependent competition in the tumour microenvironment.

We further demonstrated, using a mathematical proof, that in Yin et al.’s polymorphic exponential model^37^, the predicted time required for the tumour burden to exceed any fixed multiple of its initial value is independent of the initial size and determined solely by the fitted parameter values (Appendix 3). This structural property implies that, although the model can achieve reasonable goodness of fit to the observed data, it cannot capture treatment-dependent differences in resistance dynamics. Consequently, the polymorphic exponential model is not suitable for guiding evolutionary therapy in NSCLC. In contrast, for all two-population models deemed suitable for therapy guidance, simulations of the Zhang et al.’s evolutionary protocol^27^ predicted substantially longer times to progression compared with standard treatment.

The primary objective of evolutionary therapies is to forestall or delay the development of treatment-induced resistance. This is often done through predicting and controlling cancer cells’ response to therapy^26,32,57^. This study demonstrates that not all models are capable of capturing this. Although many models, including the Yin et al.’s polymorphic exponential model, fit the data well when the tumour burden shows a monotonic decrease, not all models can predict the U-shaped patterns typically observed when treatment-induced resistance develops. To combat resistance, being able to capture its development is vital. Therefore, though they may fit data well, models that predict long-term tumour suppression at the MTD should be approached with caution, as they may not be able to guide evolutionary therapy.

Even among the models that we found to be suitable for describing tumour dynamics in NSCLC, there are differences in the predicted time to progression (TTP). We find that the predicted TTP depends strongly on the ratio of the competition coefficients, $\frac{\alpha _{SR}}{\alpha _{RS}}$. Importantly, $\frac{\alpha _{SR}}{\alpha _{RS}} < 1$ implies that resistant cells affect the growth of sensitive cells less than sensitive cells affect resistant cells, and hence retaining a large sensitive population yields a higher TTP under Zhang et al.’s protocol and a lower TTP under MTD. Conversely, $\frac{\alpha _{SR}}{\alpha _{RS}} > 1$ implies that resistant cells have a competitive advantage over sensitive cells, resulting in a lower TTP under Zhang et al.’s protocol and a higher TTP under MTD. In models without FD, by contrast, the two populations are assumed to interact symmetrically.

Furthermore, the increase or decrease in predicted TTP can depend on the existence, reachability, and stability of interior equilibria, which are determined by the intraspecific competition coefficients^58^. In our dataset, most patients had fitted parameters such that $\frac{\alpha _{SR}}{\alpha _{RS}} > 1$. Although the median decrease in TTP predicted by models with and without frequency dependence was small, for some individuals the difference was substantial. Notably, Zhang et al.’s evolutionary therapy consistently led to longer TTP than MTD across all models, regardless of FD, underscoring the importance of accounting for asymmetric competition between sensitive and resistant cancer-cell populations.

Our study provides a methodological framework for evaluating evolutionary therapy protocols using longitudinal clinical data. By systematically comparing 26 models with varying assumptions about competition, pharmacokinetics, and treatment-induced death rate, we demonstrate how model structure shapes both goodness of fit and mechanistic interpretation. We deliberately focused on qualitative two-population models that capture population-level resistance dynamics while remaining interpretable and clinically relevant. Although the Gompertzian model with frequency-dependent competition provided the best fits for this NSCLC dataset, other cancer types, drug combinations, and treatment regimens may yield different optimal model structures. For instance, in contrast to other research^59^, we found that models fitted to this dataset did not achieve good fits under a Norton–Simon treatment-induced death rate, whereas using a log-kill assumption significantly improved goodness of fit. This is likely due to the specific drug–disease combination of NSCLC and erlotinib. We also found that including dynamic PK effects did not significantly improve the model’s goodness of fit in this dataset compared with aggregate PK.

This study is positioned within a broader landscape of alternative treatment strategies that aim to personalise cancer care and contributes to the literature on the model-informed precision dosing ^60,61^. While therapeutic drug monitoring^62^ focuses on adjusting doses to achieve target drug exposures, model-informed precision dosing uses models to adjust dosing based on patient characteristics.

Evolutionary therapies consider the ecological and evolutionary dynamics of the cancer cells^21,27,28,32,63^. The protocol simulated in this study is evolutionary as it is based on models of intra-tumoural evolution. Other approaches also based on such models include dose stabilisation^14,23,64^, intermittent dosing^16,65^, extinction therapy^66^ and the double-bind therapy^67,68^. The models used in our study may also be extended to simulate and develop these alternate strategies and/or find the best evolutionary therapy through optimisation, similarly to^69,70^. We believe that targeting resistant populations is the necessary next step in personalised therapy and will lead to patient- and treatment-specific treatment regimens which will improve patients’ quality of life and survival^26^.

Models for guiding evolutionary therapy must be based on robust patient data and incorporate key features of cancer biology, such as competition between distinct cancer cell subpopulations^23,32,58,71–73^. Bringing evolutionary therapy to clinic necessitates a collaborative approach, where pharmacologists provide insights into drug action and kinetics, mathematicians develop and refine the models, and clinical oncologists ensure biological plausibility and translate findings into patient benefit^26^. Such interdisciplinary efforts are crucial for creating models and developing therapies that will benefit patients.

Our findings emphasise the shortcomings of overtly simplistic models, such as the polymorphic exponential model, which assumes a constant mutation rate independent of treatment and neglects competition. In contrast, models with frequency-dependent selection can better capture the interplay between drug-sensitive and drug-resistant cells, improving predictive accuracy^74^. The consistent prediction across the best-fitting models that Zhang et al.’s evolutionary therapy outperforms the standard of care for all studied patients is promising. These findings provide strong theoretical support for the use of evolutionary therapy to improve care for NSCLC patients.

Our analysis indicates that, in the context of evolutionary therapy as applied in the protocol of Zhang et al. for stage IV NSCLC, the choice between dynamic and aggregate PK representations does not significantly affect the goodness of fit. Dynamic PK may, however, be important for predictions in alternative evolutionary therapy protocols, such as dose titration^14^.

In this work, we have demonstrated that evolutionary therapy can theoretically be applied in NSCLC. We showed that two-population models, which include density- and frequency-dependent competition, can capture resistance dynamics and predict treatment outcomes. These findings provide a theoretical foundation for extending evolutionary therapy to NSCLC and underscore the need for empirical validation before clinical implementation. This will require larger datasets to ensure that the data used are representative of the broader NSCLC population treated with erlotinib.

Notably, in the dataset employed here, not all patients had recorded measurements indicating tumour regrowth. Although all patients are palliative and known to progress, this event often occurs outside of the observation window. As a result, some of the models fitted to this dataset failed to predict progression. In future work, the absence of progression data could be addressed by incorporating population-level parameter estimates from progressing patients in order to constrain the range of parameter values for these patients.

The future clinical implementation of evolutionary therapy for NSCLC will require overcoming several practical challenges^26^. Validation through clinical trials will be essential to confirm the efficacy and safety of the proposed protocols. A key challenge for fast-growing cancers such as NSCLC is the need for frequent and detailed measurements of tumour burden. One promising avenue is the use of circulating tumour DNA (ctDNA) as a biomarker^75^. Collected through a simple blood sample, ctDNA is less invasive and more cost-effective than imaging, allowing for more frequent follow-up. Moreover, it can provide higher-resolution information on tumour composition, leading to more accurate and responsive model predictions. In the current study, we considered a single resistant population, which may limit predictive accuracy at the individual level, as intra-tumour heterogeneity can cause divergent responses across cancer cell types^1,3,76^. Incorporating time-series ctDNA measurements in future work will enable more dynamic, patient-specific model updates and further improve clinical relevance.

Our findings provide theoretical support for extending evolutionary therapy to NSCLC. By fitting mathematical models directly to patient data and identifying key mechanisms such as frequency-dependent selection, we offer a modelling framework to evaluate and optimise evolutionary treatment strategies. With further validation and clinical integration, these model-informed approaches may contribute to personalised regimens that improve long-term tumour control, limit resistance, and ultimately enhance patient survival and quality of life.

## Methods

### Data sources and processing

We performed our experiments using the two data sets containing patient data from the START-TKI trial (NCT05221372)^77^, previously analysed in ^37^. All patients are stage IV patients with Non-Small Cell Lung Cancer treated with erlotinib. The data includes:

1. Longest diameters of 2-7 tumours for 18 patients measured approximately every 6 weeks, with 2-18 records per patient. All 18 patients were initially given the MTD dose of 150 mg of erlotinib. Of these, 4 patients had their doses reduced to 100 mg or 50 mg due to the effects of drug-induced toxicity. For 16 of these patients, pharmacokinetic (PK) data (blood concentration levels) of erlotinib measured between 0.25 and 30.5 h after the last dose was administered. We refer to this as ‘sparsely sampled’ PK data.
2. PK data from 30 patients treated with 50–150 mg erlotinib daily with blood samples collected before treatment initiation and at 0.5, 1, 1.5, 2, 2.5, 3, 3.5, 4, 6, 8, 12, and 24 h after drug administration. We refer to this as ‘intensively sampled’ PK data.

From the tumour measurement data (18 patients, 2-7 tumours, 2-18 time points), we removed measurements from dates with some of the tumours unmeasurable. For the purpose of fitting the data to ordinary differential equation models, we also excluded patients with fewer than 6 measurements in total. We converted the longest tumour diameter measurements to volumes by assuming the tumour to be spherical. For each patient, we summed up the volumes of all their tumours to obtain their total tumour burden at each measurement date. This left a final tumour volume data set consisting 13 patients with total tumour burden information for 6-18 time points. PK data was processed by binning the sparsely sampled data into intervals of 0.5–3 h before merging with the intensively sampled PK dataset. This larger dataset was then used to create a population-level PK model.

### Ethics statement

This study involved secondary analyses of individual-level data from the START-TKI trial (ClinicalTrials.gov ID: NCT05221372). The data themselves have not been published, although analyses based on them have been previously reported (Yin et al., *CPT: Pharmacometrics & Systems Pharmacology*, 2024; 13(4): 612–623. https://doi.org/10.1002/psp4.13105). The data were obtained under a data use agreement with the original investigators at Erasmus Medical Center.

The START-TKI trial was conducted in accordance with the Declaration of Helsinki and all relevant guidelines and regulations. Ethical approval for the trial was granted by the Medical Ethics Review Committee (METC) of Erasmus Medical Center, in accordance with the Dutch Medical Research Involving Human Subjects Act (WMO), as registered on Onderzoekmetmensen.nl and ClinicalTrials.gov (NCT05221372). Informed consent was obtained from all participants prior to enrolment in the trial.

All data used in this study were de-identified prior to access, and no additional ethical approval was required for this secondary analysis.

### Pharmacokinetics

Pharmacokinetics (PK) of a drug describes how its concentration in the blood changes over the drug dosing interval. In the data we use in this study, patients typically took erlotinib once a day, resulting in a 24-h cycle in the medication dose dynamics. To study any effects drug concentration dynamics may have on tumour dynamics and our predictions, we investigate versions of each model with agPK and dyPK, explained in detail in the section titled ‘Pharmacokinetics (PK) and pharmacodynamics (PD)’ in the Results. For dyPK, the plasma concentration of the drug (in $\mu g/L$) at time *t* given a dose *D*(*t*) (in *mg*) is given by $1000\cdot D(t) \cdot C(t)$, where *C*(*t*) describes the dynamics of the plasma concentration according to a two-compartment PK model with first-order absorption and lag time, similar to Yin et al.’s study^37^ (see Appendix 2 for details). The dose *D*(*t*) is multiplied by 1000 to convert it to $\mu g$ from *mg*. This can also be written as $m(t) \cdot D_{MTD} \cdot C(t)$ where *m*(*t*) is the medication dose ratio and $D_{MTD}$ is the maximum tolerable dose in *mg*.

For agPK models, we assume that the entire dose is absorbed and the concentration stays constant, and thus $c(t)=m(t)\cdot D_{MTD} \cdot C(t) = m(t) \cdot D_{MTD}$.

The fitting procedure used for the PK model is explained in detail in Appendix 2. Of the six parameters of the model, four parameters ($V_1, CL, k_a, t_{lag}$) were fitted and two parameters were fixed ($V_2, Q$), based on the findings of Yin et al.^37^, due to their insensitivity to small changes.

### Model fitting and parameter estimation

To investigate suitable models for use in evolutionary therapy, we fitted the data of 13 patients to two-population mathematical models. We considered three standard models, with two treatment-induced death functions from the mathematical oncology literature, two pharmacodynamic options, and two options regarding frequency dependence, bringing us to 24 models (see section titled ‘Mathematical Models’ in the Results). We compared the fits of these models to the model of Yin et al., with both agPK and dyPK.

### Parameter optimisation using differential evolution

We fitted the tumour volume data to all model combinations (see section titled ‘Mathematical Models’ in the Results) as well as to the polymorphic exponential model of Yin et al. The total initial tumour burden was defined as $x_{total}(0) = x_S(0) + x_R(0)$, with the initial population set to $x_R(0)= 5\cdot 10^{-6}\cdot x_{total}(0)$ and $x_S(0) = x_{total}(0)-x_R(0)$ .

The polymorphic exponential model as described by Yin et al., contains no initial resistant population. Instead, all resistance occurs through mutation. As mutation rate is constant and independent of medication, the structure of this polymorphic exponential model means that the resistant population is initiated immediately and always grows from the start of the simulation. Consequently, the dynamics of the model are not substantially changed by our including a small initial population of resistant cells.

The remaining model parameters were estimated using Python 3.11.4 with the differential evolution algorithm^54^ implemented in the scipy.optimize library. Differential evolution (DE) is a global optimisation algorithm designed for continuous, non-linear problems. It evolves a population of candidate parameter vectors through mutation, recombination, and selection^54^. In each generation, parameter vectors are randomly perturbed and recombined to explore the parameter space. The recombined vector’s cost (or goodness of fit in the context of this paper) is compared to the target vector’s cost, and the vector with the lower cost is retained for the next generation. The optimization process terminates when the standard deviation of the population’s costs falls below $r_{\text {tol}} \cdot |\text {mean}(\text {costs})|$, where $r_{\text {tol}}$ is the relative tolerance. This convergence criterion ensures the population stabilises and no significant improvements are achievable. The model was fitted to the data by solving the differential equations with the Runge–Kutte 45 method from the solve_ivp module in the scipy.integrate library. Although DE is stochastic and explores a large portion of the parameter space on a single run, we ran the fitting procedure five times per patient and selected the result with the lowest error. We used the default settings for differential evolution, except for decreasing relative tolerance to $1 \times 10^{-7}$.

#### Parameter constraints

We fit the model parameters through differential evolution, applying bounds summarised in Table 3. The bounds for the growth rates $r_{R}$ and $r_{S}$ based on the literature. For this patient data set, Yin et al reported upper and lower bounds for the growth in tumour’s longest diameter. Converting diameter to tumour volume (assuming tumour is spherical) gave upper and lower bounds of 0.00255 and 0.0921, respectively. We also considered growth rates reported from in vitro studies, such as ^39^. This gave us growth rates being bounded between 0.01 and 0.05 for the Gompertzian and polymorphic exponential models. In the von Bertalanffy model   (Eq. 3), $r_S$ and $r_R$ are multiplied by the populations $x_S$ and $x_R$ raised to fractional powers. This results in smaller net growth rates compared to the other models. This means that the values of $r_S$ and $r_R$ need to be higher to achieve the same net growth rates of the population. For this reason, the upper bounds for $r_S$ and $r_R$ for the von Bertalanffy model were set to 0.5.

Individual bounds for the carrying capacity *K* were set between 1.5 and 5 times the initial tumour burden.

As $c(t)\in [0,D_{MTD}]$ where $D_{MTD} = 150$ mg of erlotinib, we set different upper bounds for $\lambda$, specifically 0.001 for models with LK, and 0.01 for models with NS.

The lower and upper bounds for the competition coefficients $\alpha _{_{\textrm{SR}}}$ and $\alpha _{_{\textrm{RS}}}$ were set at 0.1 and 10,  respectively. We chose to explore the high upper bound due to the findings of relatively high competition coefficients in recent studies, such as those of Freischel et al.^38^.

**Table 3:** Parameter bounds used for differential evolution fitting for individual and population fits. Units: $$r_S$$, $$r_R$$, $$\mu$$ – day$$^{-1}$$; $$\lambda$$ – mg$$^{-1}$$day$$^{-1}$$; *K*, $$x_S(t)$$, $$x_R(t)$$ – mm$$^3$$; $$\alpha _{SR}$$, $$\alpha _{RS}$$ – dimensionless (denoted as “–”). Abbreviations: GM – Gompertzian, LG – Logistic, Exp – Exponential, LK – log kill, NS – Norton Simon, FD – Frequency dependence, agPK – aggregated pharmacokinetics, dyPK – dynamic pharmacokinetics. Here, $v(0)$ refers to the initial tumour burden. Specifically, $v(0) = x_{total}(0)$ in the case of individual fits, and $v(0) = max(x_{total}_1(0), x_{total}_2(0)..., x_{total}_13(0))$ (highest initial tumour burden among the 13 patients) in the case of population fits.

Models	$r_S$ (day$^{-1}$)	$r_R$ (day$^{-1}$)	$\lambda$ (mg$^{-1}$day$^{-1}$)	*K* (mm$^3$)	$\alpha _{SR}$ (-)	$\alpha _{RS}$ (-)
GM LK FD agPK/dyPK; LG LK FD agPK/dyPK	[0.001, 0.1]	[0.001, 0.1]	[0.00001, 0.001]	$[1.5\,v(0), 5\,v(0)]$	[0.1, 10]	[0.1, 10]
GM LK noFD agPK/dyPK; LG LK noFD agPK/dyPK	[0.001, 0.1]	[0.001, 0.1]	[0.00001, 0.001]	$[1.5\,v(0), 5\,v(0)]$	n.a.	n.a.
GM NS FD agPK/dyPK; LG NS FD agPK/dyPK	[0.001, 0.1]	[0.001, 0.1]	[0.00001, 0.01]	$[1.5\,v(0), 5\,v(0)]$	[0.1, 10]	[0.1, 10]
GM NS noFD agPK/dyPK; LG NS noFD agPK/dyPK	[0.001, 0.1]	[0.001, 0.1]	[0.00001, 0.01]	$[1.5\,v(0), 5\,v(0)]$	n.a.	n.a.
VB LK/NS FD agPK/dyPK	[0.001, 0.5]	[0.001, 0.5]	[0.00001, 0.01]	$[1.5\,v(0), 5\,v(0)]$	[0.1, 10]	[0.1, 10]
VB LK/NS noFD agPK/dyPK	[0.001, 0.5]	[0.001, 0.5]	[0.00001, 0.01]	$[1.5\,v(0), 5\,v(0)]$	n.a.	n.a.
Parameters Yin et al. model	*r*	$\mu$	$\lambda$			
Bounds Yin et al. model	[0.00001, 0.05]	[0.00005, 0.05]	[0.000001, 0.05]			

#### Individual and population-level parameter estimation

Each model was fitted for each patient using differential evolution to minimise the following cost function:

8 $$\begin{aligned} \text {Individual level relative percentage error }= \left( \frac{RMSE}{v_i(0)} \right) \cdot 100. \end{aligned}$$

Here, $v_i(0)$ is the total tumour volume of the *i*th patient at the start of treatment, and RMSE is the root mean squared error. We converted RMSE to relative percentage error in order to simplify comparison of fits between patients with different total tumour burdens. By minimizing this cost function, we determined the individual best-fit parameters for each patient.

Although we have confirmation that all patients in the dataset progressed, this is often not reflected in the data. We have also observed that the earlier and later data points are most important for capturing the trend. When, as occurs in our data, the last data points do not indicate progression, it is difficult for an individual model to predict the tumour growth trajectory accurately, even though many models may fit the data well.

To account for this, we additionally sought for each model combination a single set of parameters that would fit best to the all individual patients, given the unique initial tumour burden of each patient. We fitted a cost function for population-level relative percentage error which calculates the error as a percentage of the patient’s initial tumour burden for each patient, then averages this over the population (Eq. 9).

9 $$\begin{aligned} \text {Population-level relative percentage error }= \frac{1}{n}\sum ^n_{i=1}\left( \frac{RMSE_i}{v_i(0)} \right) \cdot 100. \end{aligned}$$

In this equation, *n* is the number of patients, $v_i(0)$ and $RMSE_i$ are the initial tumour volume and root mean squared error of the *i*th patient respectively.

#### Generalizing model fits across patients

**Fig. 12:**
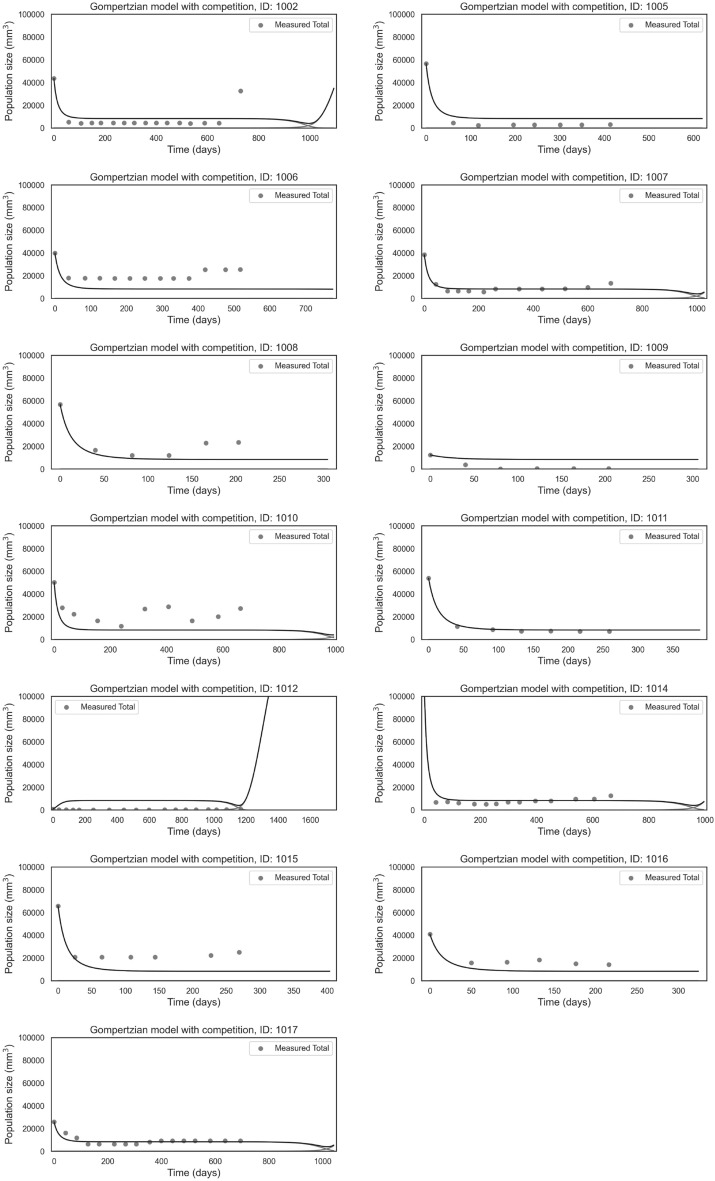
Population fits for the Gompertzian model with frequency dependence and the log-kill treatment-induced death rate. The population fits show the same parameter set fitted to all patients, with each patient having their own initial tumour burden.

In the population fitting we sought a single set of parameters that would give the lowest error across all patients, given their individual starting size. The results from the population fitting are shown in Table 2, and it can be seen that the Gompertzian model with fitted competition coefficients fits best to all patients with a relative percentage error of $5.7\%$ with and without PK. The fits of this model to all patients in the population are shown in Fig. 12.

### Simulation of evolutionary therapy protocols

We used the models selected in the previous step to compare the predicted time before the tumour first exceeds 1.1 times its original size (time to progression) under the MTD treatment protocol with that under the evolutionary protocol of Zhang et al.^78^. The Zhang et al.’s protocol consists of two phases: with and without medication. With medication, the tumour growth is simulated using the optimised parameters for the selected model, with the dose ratio *m*(*t*) set to 1. Without medication, the medication dose is set to 0, keeping all other parameters the same.

Using the selected ordinary differential equations population dynamics, the simulation is initialised with medication on phase, starting with the provided initial total tumour burden. When the total predicted tumour burden decreases to $\le 50\%$ of its initial value, the medication off phase commences. When the total predicted tumour burden returns to its initial value, then the medication on phase resumes. This cycle continues for the desired length of the simulation. To measure time to progression we defined progression as 1.1 times the initial estimated sum of tumour volumes. For each patient, we used the parameters found in the fitting process to simulate future tumour growth and calculate the predicted time to progression under the MTD and Zhang et al.’s protocols.

The impact of evolutionary therapy on all models was assessed using Kaplan-Meier survival plots, where the event of interest is TTP. A Kaplan-Meier plot is a non-parametric approach to survival analysis which allows the comparison of time-to-event data between different groups. Both MTD and evolutionary therapy scenarios were used to simulate tumour volume over a period 10 times the original duration. Patients who did not progress by the end of this period were assigned to the longest time to progression. We employed the log-rank test to compare survival distributions of the MTD and evolutionary therapy groups. As the log-rank test accounts for censored data, the log-rank test statistic, which follows a chi-squared distribution, provides an unbiased comparison of observed versus expected outcomes between groups.

## Figures and Tables

**Fig. 7:**
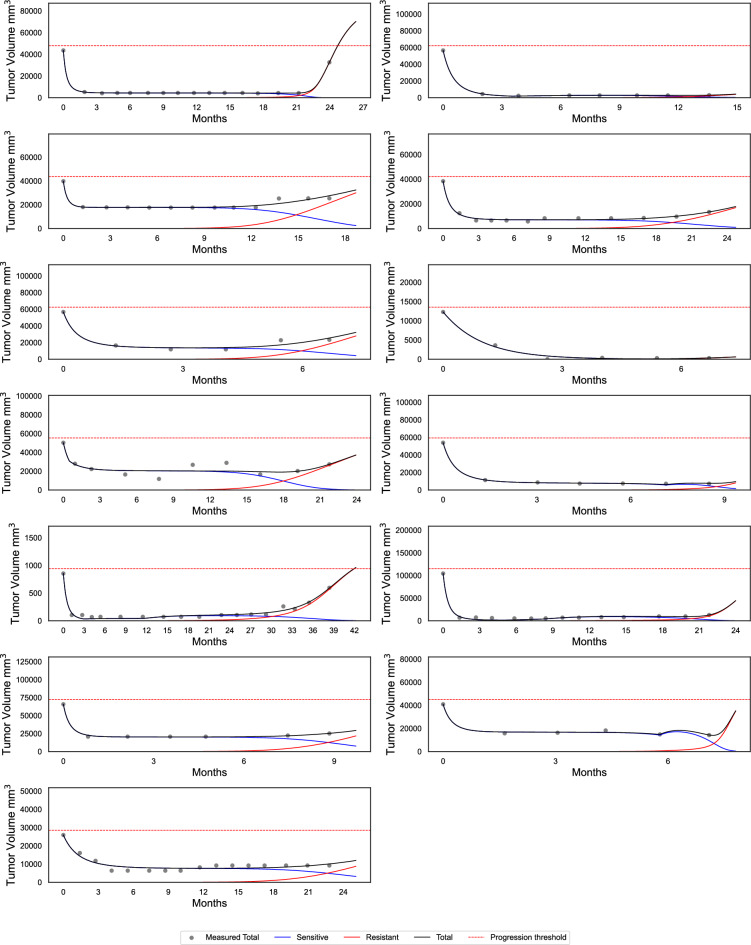
All individual fits for the Gompertzian model with frequency dependence, the log-kill treatment-induced death rate, and without pharmacodynamics. On some patients changes in dose cause the line to abruptly change direction.

**Fig. 8:**
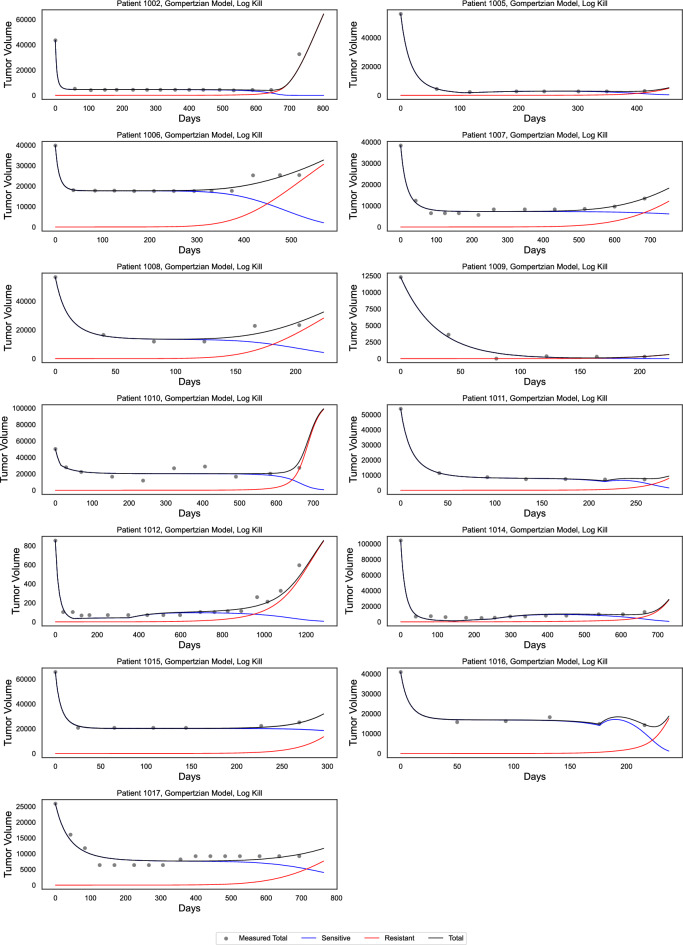
All individual fits for the Gompertzian model with frequency dependence, log-kill and pharmacokinetics.

**Fig. 9:**
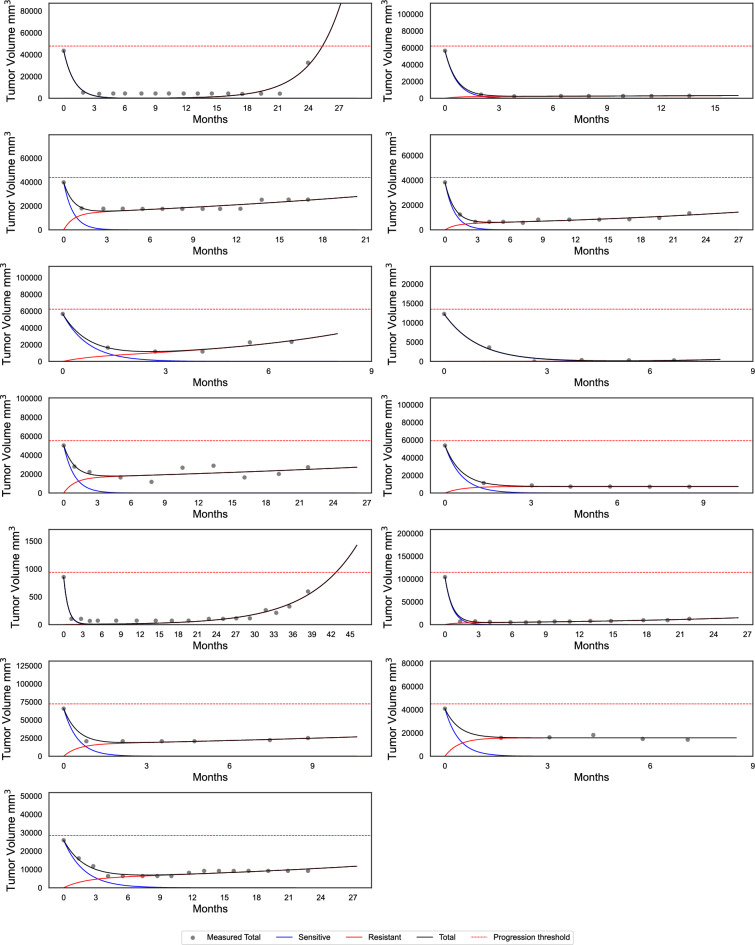
All individual fits for the exponential model. In many patients a period of initial fast growth occurs due to a very high rate of mutation meaning growth slows once all sensitive cells have mutated.

**Fig. 10:**
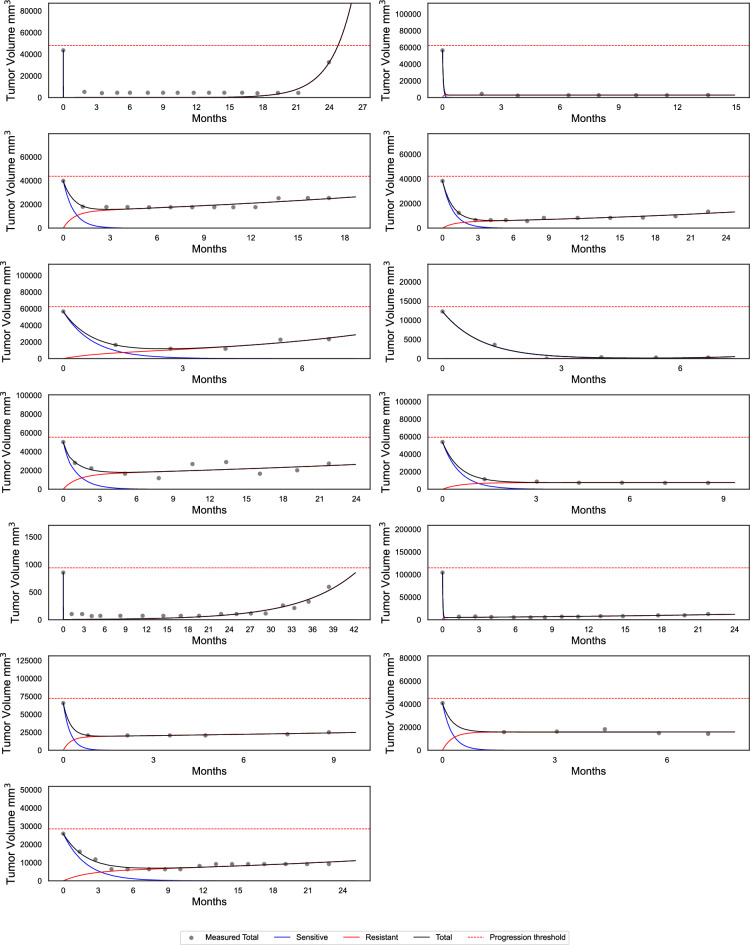
All individual fits for the exponential model with pharmacokinetics. In many patients a period of initial fast growth occurs due to a very high rate of mutation meaning growth slows once all sensitive cells have mutated.

**Fig. 13:**
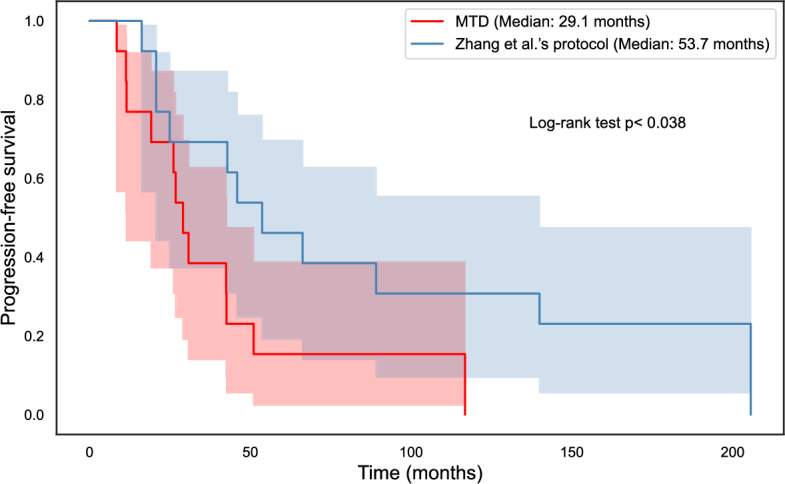
Kaplan-Meier plot showing simulated progression-free survival for individually fitted Gompertzian model without frequency dependence, with the log-kill treatment-induced death rate and agPK.

**Fig. 14:**
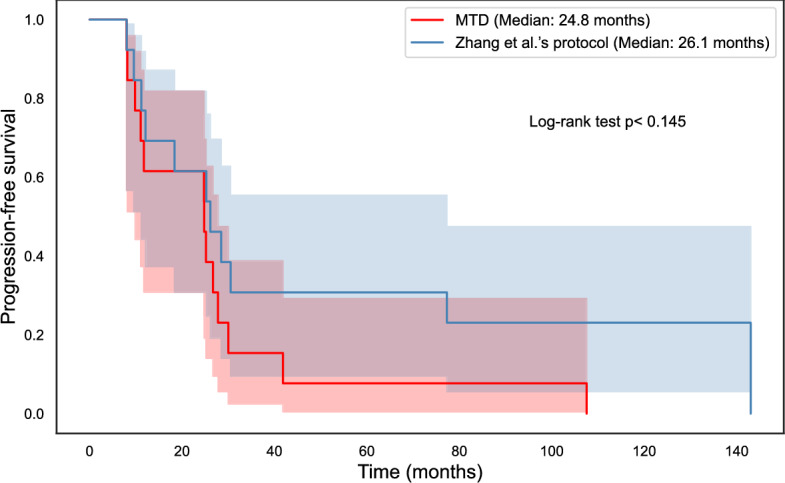
Kaplan-Meier plot showing simulated progression-free survival for individually fitted Logistic model with frequency dependence, with the log-kill treatment-induced death rate and agPK.

**Fig. 15:**
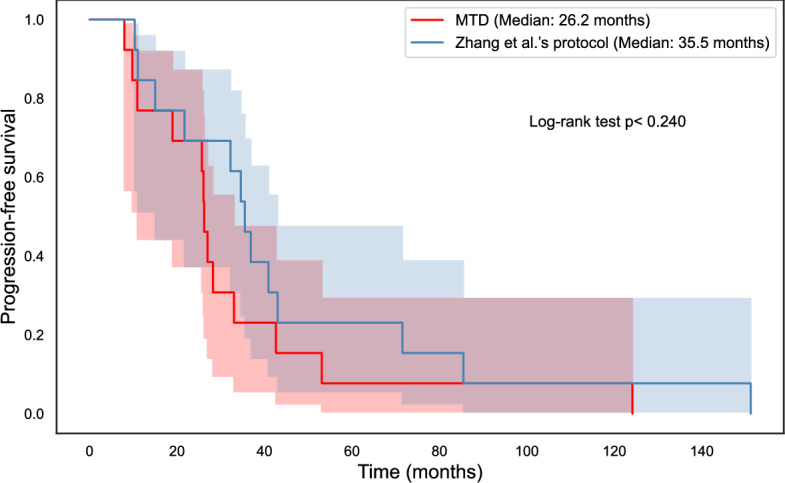
Kaplan-Meier plot showing simulated progression-free survival for individually fitted Logistic model without frequency dependence, with the log-kill treatment-induced death rate and agPK.

## Data Availability

The data from the START-TKI clinical trial (ClinicalTrials.gov ID: NCT05221372) consist of individual patient records, which are described in detail in the Methods section. Due to privacy and ethical restrictions, these individual-level data are not publicly available. However, they may be made available upon reasonable request to Anne-Marie Dingemans at Erasmus Medical Center, subject to institutional and ethical approvals. Metadata and the code used for data fitting, plotting, and simulation are available at: https://gitlab. tudelft.nl/evolutionary-game-theory-lab/erlotinib_project.

## Appendix 1: Clinical trials on evolutionary therapy

This appendix provides an overview of clinical trials studying the use of evolutionary therapy in various cancers. These trials explore treatment strategies that adjust drug dosing based on tumour response to delay resistance and improve patient outcomes. Table 4 summarises key studies, including trial locations, cancer types, and registration numbers.

**Table 4:** Key clinical trials on evolutionary therapy.

Study	Location	Cancer type	Trial number
A Pilot Study of Adaptive Abiraterone Therapy for Metastatic Castration Resistant Prostate Cancer	H. Lee Moffitt Cancer Center and Research Institute	Metastatic Castrate Resistant Prostate Cancer	NCT02415621
ANZadapt: Phase II Randomised Controlled Trial of Patient-specific Adaptive Versus Continuous Abiraterone or Enzalutamide in Metastatic Castration-resistant Prostate Cancer	Leiden University Medical Center	Metastatic Castrate Resistant Prostate Cancer	NCT05393791
A Phase IIA Study of Sequential (“First Strike, Second Strike”) Therapies, Modeled on Evolutionary Dynamics of Anthropocene Extinctions, for High Risk Metastatic Castration Sensitive Prostate Cancer	H. Lee Moffitt Cancer Center and Research Institute	High Risk Metastatic Castrate Sensitive Prostate Cancer	NCT05189457
A Phase 1b Study of Adaptive Androgen Deprivation Therapy for Stage IV Castration Sensitive Prostate Cancer	H. Lee Moffitt Cancer Center and Research Institute	Stage IV Castration Sensitive Prostate Cancer	NCT03511196
Adaptive Therapy of Vismodegib in Advanced Basal Cell Carcinoma	H. Lee Moffitt Cancer Center and Research Institute	Advanced Basal Cell Carcinoma	NCT05651828
First Strike, Second Strike Therapies for High Risk Metastatic Castration Sensitive Prostate Cancer	H. Lee Moffitt Cancer Center and Research Institute	High Risk Metastatic Castrate Sensitive Prostate Cancer	NCT05189457
Intermittent Androgen Deprivation Therapy for Stage IV Castration Sensitive Prostate Cancer	H. Lee Moffitt Cancer Center and Research Institute	Stage IV Castration Sensitive Prostate Cancer	NCT03511196
Adaptive Chemotherapy for Ovarian Cancer in Patients With Relapsed Platinum-sensitive High Grade Serous or High Grade Endometrioid Ovarian Cancer	University College London	Platinum-sensitive High Grade Serous or High Grade Endometrioid Ovarian Cancer	NCT05080556
Adaptive BRAF-MEK Inhibitor Therapy for Advanced BRAF Mutant Melanoma	H. Lee Moffitt Cancer Center and Research Institute	BRAF Mutant Melanoma	NCT03543969
Evolutionary Inspired Therapy for Newly Diagnosed, Metastatic, Fusion Positive Rhabdomyosarcoma	H. Lee Moffitt Cancer Center and Research Institute	Newly Diagnosed, Metastatic, Fusion Positive Rhabdomyosarcoma	NCT04388839
Generating Novel Therapeutic Strategies Based on Evolutionary Tumor Board	H. Lee Moffitt Cancer Center and Research Institute	Various	NCT04343365
Generating Novel Translational and Therapeutic Strategies Based on a Multicenter, Pediatric and AYA Evolutionary Tumor Board	H. Lee Moffitt Cancer Center and Research Institute	Various	NCT06423950

## Appendix 2: Pharmacokinetic modelling

This appendix describes how we incorporated pharmacokinetics (PK) into our model to account for drug absorption, distribution, and elimination in the body. We used a two-compartment model with first-order absorption and linear elimination to represent drug concentration changes over time. The equation below gives the time-varying drug concentration, *C*(*t*), in the central compartment, normalised to the treatment dose^79^:

10 $$\begin{aligned} C(t) = {\left\{ \begin{array}{ll} \frac{A e^{-\alpha (t - t_D + \tau - t_{\text {lag}})}}{1 - e^{-\alpha \tau }} + \frac{B e^{-\beta (t - t_D + \tau - t_{\text {lag}})}}{1 - e^{-\beta \tau }} - \frac{(A + B) e^{-k_a (t - t_D + \tau - t_{\text {lag}})}}{1 - e^{-k_a \tau }} & \text {if } t - t_D < t_{\text {lag}}, \\ \frac{A e^{-\alpha (t - t_D - t_{\text {lag}})} }{1 - e^{-\alpha \tau }} + \frac{B e^{-\beta (t - t_D - t_{\text {lag}})}}{1 - e^{-\beta \tau }} - \frac{(A + B) e^{-k_a (t - t_D - t_{\text {lag}})}}{1 - e^{-k_a \tau }} & \text {if not,} \end{array}\right. } \end{aligned}$$

where

$$A = \frac{\frac{k_a}{V_1} \left( \frac{Q}{V_2} - \alpha \right) }{(k_a - \alpha )(\beta - \alpha )}, B = \frac{\frac{k_a}{V_1} \left( \frac{Q}{V_2} - \alpha \right) }{(k_a - \beta )(\alpha - \beta )},$$

and

$$\alpha = \frac{\frac{Q}{V_2} \frac{CL}{V_1}}{\beta }, \quad \beta = \frac{1}{2} \left[ \frac{Q}{V_1} + \frac{Q}{V_2} + \frac{CL}{V_1} - \sqrt{\left( \frac{Q}{V_1} + \frac{Q}{V_2} + \frac{CL}{V_1} \right) ^2 - 4 \frac{Q CL}{V_2 V_1}} \right].$$

Here, $t_D$ is the time since the last dose (in hours), $\tau$ is the time between consecutive doses (24 h), $t_\textrm{lag}$ is the absorption lag time, $k_a$ is the absorption rate constant, Q is the apparent distribution clearance rate (L/h), CL is the apparent clearance rate (L/h), and V_1_ and V_2_ are apparent distribution volumes (L) of the central and peripheral compartments, respectively.

Of the six parameters of the model (V_1_, V_2_, Q, CL, k_a_, t_lag_), four (V_1_, CL, k_a_, t_lag_) were fitted and two (V_2_, Q) were fixed based on the findings of a previous study^37^ due to their insensitivity to small changes. Bioavailability was set to 1, consistent with previous studies^37^.

The bounds used for fitting the four parameters are in Table 5.

**Table 5:** Bounds for the fitted parameters of the PK model.

Parameter	V_1_ (L)	CL (L/h)	k_a_ (/h)	t_lag_ (h)
**Bounds**	10 - 300	0.1 - 8	0.1 - 5	0.1 - 0.6

The PK data consisted of sparsely and intensively sampled data as described in the Methods section. The concentrations for all patients were normalised by dose and data from all 46 patients were pooled. A bootstrapping approach was used, with 1000 iterations involving random sampling of 46 patients with replacement. The PK model was fit to each resampled dataset. This process allowed for the assessment of variability and robustness of the population parameter estimates.

The fitting procedure used the ‘scipy.minimize’ python package with the ‘L-BFGS-B’ solver and root mean squared error (RMSE) as the cost function, testing 1000 random initial guesses to ensure reliable parameter estimation.

Table 6 shows the mean and standard deviation of the fitted parameter values. These parameter values were used to simulate pharmacokinetics for all subsequent analyses. While individual variability in PK parameters was recognised, using population parameters was considered appropriate given the goal of capturing overall treatment dynamics rather than precise individual-level variation, which is less critical in long-term cancer therapy simulations.

**Table 6:** Mean fitted parameter values for the PK model.

Parameter	V_1_ (L)	CL (L/h)	k_a_ (/h)	t_lag_ (h)
**Mean value (SD)**	192.78 (37.04)	4.42 (0.23)	2.75 (0.64)	0.45 (0.09)

It is important to note that in this study, we fit the PK model using a custom implementation in Python, employing the integrated form of the model equations (Eq. 10, Appendix 2) and optimising parameters with standard functions from the Scipy package^80^. While it is common practice in the field to use specialised software such as NONMEM for PK modelling^81^, we deliberately chose a manual implementation to ensure full transparency and control over all modelling assumptions. This includes explicit specification of the cost function, numerical solvers, and integration tolerances. Although tools such as NONMEM are powerful and widely adopted, they often function as black boxes with internal implementation details such as cost functions, step sizes, algorithmic heuristics, or convergence criteria-remaining unclear to users outside the field. In contrast, our approach promotes reproducibility and transparency, allowing all modelling choices to be fully documented and scrutinised.

## Appendix 3: Time to progression in the polymorphic exponential model is independent of the tumour burden

The polymorphic exponential model used by Yin et al. is defined as

11 $$\begin{aligned} {\left\{ \begin{array}{ll} \dot{x}_S & = (r-\lambda -\mu ) x_S, \\ \dot{x}_R & = r x_R + \mu x_S, \end{array}\right. } \end{aligned}$$

with *r* the growth rate, $\lambda$ the effect of medication, and $\mu$ the mutation rate of sensitive to resistant cells.

In^37^ the polymorphic exponential model has the initial condition $x_R(0) = 0$. Denoting the total tumour burden at time *t* by $x_{total}(t),$ so that $x_{total}(t) = x_S(t) + x_R(t),$ we examine a more general case, where initial sensitive $x_S(0)$ and resistant $x_R(0)$ populations are each proportional to the initial tumour burden $x_{total}(0)$. That is, we assume

$$\begin{aligned} x_S(0)&= (1 - \kappa )x_{total}(0),\\ x_R(0)&= \kappa x_{total}(0). \end{aligned}$$

with $\kappa \in [0,1].$ For simplicity of calculations, let us re-express the first initial condition, so that

$$x_{total}(0) = \dfrac{1}{1-\kappa } x_S(0).$$

Then, letting $k = \frac{\kappa }{1-\kappa }$, it follows that

12 $$\begin{aligned} {\left\{ \begin{array}{ll} x_S(0) & = x_S^0,\\ x_R(0) & = k x_S^0. \end{array}\right. } \end{aligned}$$

We now will solve for the analytical solution to Eq. 11 with the initial conditions given by Eq. 12. In particular, we are interested in using the solution to find information about the time to progression, when the total population reaches some specified multiple of the initial tumour burden (i.e. the time at which $x_{total}(t) = p x_{total}(0)$). Expressed in matrix-vector form, the system becomes

$$\begin{aligned} \frac{d}{dt}\begin{bmatrix} x_S\\ x_R\\ \end{bmatrix} = \begin{bmatrix} r-\lambda -\mu & & \quad 0 \\ \mu & & \quad r\\ \end{bmatrix} \begin{bmatrix} x_S\\ x_R \end{bmatrix}. \end{aligned}$$

The eigenvalues of the matrix are $r-\lambda -\mu$ and *r*. We must now find the eigenvector associated with each.

**Eigenvector** $\textbf{v}_{1}$ **for eigenvalue** $r-\lambda -\mu$: We must find a vector $\textbf{v}_1$ in the null space of

$$\begin{bmatrix} 0& \quad 0 \\ \mu & \quad \lambda +\mu \end{bmatrix}.$$

To this end, find $v_1^1$ and $v_1^2$ such that:

$$\begin{aligned}&\begin{bmatrix} 0& \quad 0 \\ \mu & \quad \lambda +\mu \end{bmatrix} \begin{bmatrix} v^1_1\\ v^2_1 \end{bmatrix} = \begin{bmatrix} 0\\ 0 \end{bmatrix}. \end{aligned}$$

It follows that $v^2_1 = \left( \frac{-\mu }{\lambda + \mu }\right) v^1_1$ and $\textbf{v}_1$ can be taken to be

$$\begin{aligned} \textbf{v}_1 = \begin{bmatrix} \textbf{1}\\ \left( \frac{-\mu }{\lambda + \mu }\right) \end{bmatrix}. \end{aligned}$$

**Eigenvector** $\textbf{v}_2$ **for eigenvalue** $r$: Similar calculations follow for eigenvalue $r$, so that we must find $v_2^1$ and $v_2^2$ satisfying:

$$\begin{aligned} \begin{bmatrix} -\lambda -\mu & \quad 0 \\ \mu & \quad 0 \end{bmatrix} \begin{bmatrix} v_2^1\\ v_2^2 \end{bmatrix} = \begin{bmatrix} 0\\ 0 \end{bmatrix} \end{aligned}$$

Thus, $\mu v^1_2=0$ and $(-\lambda -\mu )v_2^1=0$, implying that $v_2^1 = 0$ and $v_2^2$ is free, so $$\begin{bmatrix} 0\\ 1 \end{bmatrix}$$ is an eigenvector.

**The solution**: We can construct the general solution from these eigenvalue and eigenvector pairs:

$$\begin{aligned} \begin{bmatrix} x_S(t)\\ x_R(t) \end{bmatrix} =c_1 e^{(r-\lambda -\mu )t} \begin{bmatrix} 1\\ \left( \frac{-\mu }{\lambda + \mu }\right) \end{bmatrix} + c_2e^{rt}\begin{bmatrix} 0\\ 1 \end{bmatrix}. \end{aligned}$$

Using initial conditions from (12), $x_S(0)=x^0_S$ and $x_R(0)=k x^0_S$, we can determine $c_1$. Since

$$\begin{aligned} x_S(t) = c_1 e^{(r-\lambda -mu)t}, \end{aligned}$$

it follows that $c_1 = x^0_S$. Considering the second equation,

$$\begin{aligned} x_R(t)&= c_1e^{(r-\lambda -\mu )t}\left( \frac{-\mu }{\lambda +\mu }\right) + c_2 e^{rt}\\&= x_S^0 e^{(r-\lambda -\mu )t}\left( \frac{-\mu }{\lambda +\mu }\right) + c_2 e^{rt}. \end{aligned}$$

From (12), $x^0_R = kx^0_S$, so that

$$\begin{aligned} x_R(0) = kx^0_S=\left( \frac{-\mu }{\lambda +\mu }\right) x^0_S + c_2 \end{aligned}$$

and $c_2 = (k + \frac{\mu }{\lambda +\mu })x^0_S.$ This leads to the solution:

13 $$\begin{aligned} {\left\{ \begin{array}{ll} x_S(t)=x^0_S e^{(r-\lambda -\mu )t},\\ x_R(t) = x^0_S\left( \frac{-\mu }{\lambda +\mu }\right) e^{(r-\lambda -\mu )t}+ x_S^0(k+\frac{\mu }{\lambda +\mu })e^{rt}. \end{array}\right. } \end{aligned}$$

**Time to Progression**: We now consider how the total tumour burden develops over time relative to the initial tumour burden. To this end, let us calculate the total tumour burden at time *t*:

14 $$\begin{aligned} x_S(t) + x_R(t) = x_S^0 e^{(r-\lambda -\mu )t} + x_S^0 \left( \frac{-\mu }{\lambda +\mu }\right) e^{(r-\lambda -\mu )t}+x_S^0\left( k+\frac{\mu }{\lambda +\mu }\right) e^{rt}. \end{aligned}$$

Time to progression is calculated as the time at which the total tumour burden (Eq. (14)) reaches some multiple of the initial tumour burden

15 $$\begin{aligned} p \, x_{total}(0)=p (x_S^0+x_R^0)=(1+k)p x_S^0. \end{aligned}$$

One can see that when equating (14) to (15), the $x_S^0$ will cancel, so that

$$\begin{aligned} p&= \frac{1}{1+k}\left( e^{(r-\lambda -\mu )t} - \frac{\mu }{\lambda +\mu }e^{(r-\lambda -\mu )t}+\left( k+\frac{\mu }{\lambda +\mu }\right) e^{rt}\right) . \end{aligned}$$

Therefore, for this model with initial conditions given by Eq. (12), the time at which the tumour burden reaches any multiple of initial burden is independent of $x_{total}(0)$, so that the time to progression does not depend on the starting population. This general proof holds for any value of *k*, including the initial conditions used by Yin et al. where $k=0$ and for our data fitting where we assume a small value of *k* to estimate $x_R^0$.

**Simulating evolutionary therapy:** Under Zhang et al.’s protocol, treatment is switched between on and off depending on the tumour burden. Evolutionary therapy is simulated under this protocol with the parameters derived from the fitted models. To preserve a sufficient sensitive cell population to control the resistant, a lower limit is set at 50% of the initial tumour burden. Medication is paused when the total tumour burden has declined to this level, i.e., when $x_{total} \le 0.5\cdot x_{total}(0)$. Treatment is resumed once the tumour burden returns to its initial size, i.e., when $x_{total} \ge x_{total}(0)$. These cycles continue as long as a large enough sensitive cell population remains to respond to medication.

We have shown above that for the polymorphic exponential model, for any given set of parameters, the time to reach $0.5\cdot x_{total}(0)$ is the same for all values of $x_{total}(0)$. When medication is paused, the time for the tumour burden to return to its initial size is likewise the same for all values of $x_{total}(0)$ as we have also shown that the medication effect $\lambda$ is independent of $x_{total}(0)$. This means that the timing of the medication state changes and the subsequent time to progression happen at the same times for any $x_{total}(0)$. In the polymorphic exponential model, the mutation effect $\mu$ is independent of $\lambda$. This means that resistant population increases in proportion to the size of the sensitive population, independent of the presence or absence of medication. Consequently, when medication is paused, the sensitive population increases, and this in turn increases the growth rate of the resistant cells. The polymorphic exponential model therefore predicts that resistant cells grow faster in the absence of medication, and evolutionary therapy may give a worse outcome than MTD.

## Appendix 4: Individual fits

This appendix presents the results of fitting different tumour growth models to individual patient data. By comparing model predictions to actual tumour measurements, we evaluated how well each model captured changes in tumour size over time. The analysis includes fitting models such as the Gompertzian, Logistic, von Bertalanffy, and exponential growth models, along with variations that incorporate different treatment-induced death rates (log-kill and Norton-Simon) and competition coefficients. We also incorporated pharmacokinetics in some models to account for how the drug concentration changes in the body, Figs. 7, 8, 9 and 10. The results showed that including pharmacokinetics had minimal impact on the model’s accuracy. Box plots comparing the mean percentage error for all tested models indicated that the Gompertzian model with frequency dependency consistently performed the best, Fig. 11. It also includes the results of Welch’s t-test comparing models with and without pharmacokinetics. We can see from these results that there is no significant difference in error for models with and without PK (Table 7).

## Appendix 5: Population-wide fits

In addition to individual model fits, we assessed how well each model performed when applied to the entire patient group. Instead of fitting parameters separately for each patient, we used a single set of parameters across all patients to identify general tumour growth patterns. The results show that the Gompertzian model with frequency dependence provides the best overall fit. Other models, such as the logistic and von Bertalanffy models, did not fit as well (Fig. 12).

## Appendix 6: Statistical analysis

For the individual fits, we conducted paired t-tests, using the Bonferroni correction for multiple samples, to see if the error was affected by the type of pharmacokinetics used in the model. For all best-fitting models the effect size was negligible, meaning there was < 1% difference in error. The logistic model without frequency dependence did show a significant difference between aggregate pharmacokinetics and dynamic pharmacokinetics, with an effect size <0.1%. The 24 h cycle of the pharmacodynamics means that small changes in the lambda parameter may adjust the height of the wave, giving miniscule improvements in fit without having any effect on the long term tumour growth dynamics (Table 7).

**Table 7:** Paired t-test results comparing model fits using aggregated pharmacokinetics (agPK) versus dynamic pharmacokinetics (dyPK), with multiple testing correction applied. The table reports the t-statistic, raw p-value, corrected p-value, significance status, and effect size for each model. Abbreviations: GM – Gompertzian, LG – Logistic, Exp – Exponential, LK – log kill, NS – Norton Simon, FD – Frequency dependence, agPK – aggregated pharmacokinetics, dyPK – dynamic pharmacokinetics.

Model	Paired t-stat	p-val	Corrected p-val	Significant	Effect size
Best-fitting models					
GM LK FD	-0.958205	0.356868	1.0	False	0.018636
GM LK	-3.452648	0.004782	0.06216	False	0.072227
LG LK FD	-1.698098	0.115246	1.0	False	0.03479
LG LK	-5.693077	0.0001	0.001303	True	0.076815
Exp	-0.798303	0.440208	1.0	False	0.214008
Other models					
GM NS FD	0.222386	0.827754	1.0	False	0.021366
LG NS FD	0.359027	0.725813	1.0	False	0.077937
VB LK FD	-4.057152	0.00159	0.020667	True	0.062133
VB NS FD	0.540878	0.598489	1.0	False	0.075199
GM NS	-1.205718	0.251163	1.0	False	0.10949
LG NS	-2.757242	0.017368	0.22578	False	0.11868
VB LK	-4.0286275	0.001673	0.021753	True	1.180861
VB NS	0.656555	0.523859	1.0	False	0.090505

## Appendix 7: Kaplan-Meier plots

Kaplan-Meier plots for the 3 best-fitting models not already included in the text. All predict that Zhang et al.'s evolutionary therapy will increase progression-free survival compared to MTD (Figs. 13, 14, 15).
